# Positional priming of visual pop-out search is supported by multiple spatial reference frames

**DOI:** 10.3389/fpsyg.2015.00838

**Published:** 2015-06-16

**Authors:** Ahu Gokce, Hermann J. Müller, Thomas Geyer

**Affiliations:** ^1^Department of Psychology, Kadir Has University, IstanbulTurkey; ^2^Department of Psychology, Ludwig-Maximilians-Universität München, MunichGermany; ^3^School of Psychology, Birkbeck College, University of London, LondonUK

**Keywords:** visual search, positional priming of pop-out, reference frames, spatial maps, working memory

## Abstract

The present study investigates the representations(s) underlying positional priming of visual ‘pop-out’ search (Maljkovic and Nakayama, 1996). Three search items (one target and two distractors) were presented at different locations, in invariant (Experiment 1) or random (Experiment 2) cross-trial sequences. By these manipulations it was possible to disentangle retinotopic, spatiotopic, and object-centered priming representations. Two forms of priming were tested: target location facilitation (i.e., faster reaction times – RTs– when the trial *n* target is presented at a trial *n-1* target relative to *n-1* blank location) and distractor location inhibition (i.e., slower RTs for *n* targets presented at *n-1* distractor compared to *n-1* blank locations). It was found that target locations were coded in positional short-term memory with reference to both spatiotopic and object-centered representations (Experiment 1 vs. 2). In contrast, distractor locations were maintained in an object-centered reference frame (Experiments 1 and 2). We put forward the idea that the uncertainty induced by the experiment manipulation (predictable versus random cross-trial item displacements) modulates the transition from object- to space-based representations in cross-trial memory for target positions.

## Introduction

**FIGURE 1:**
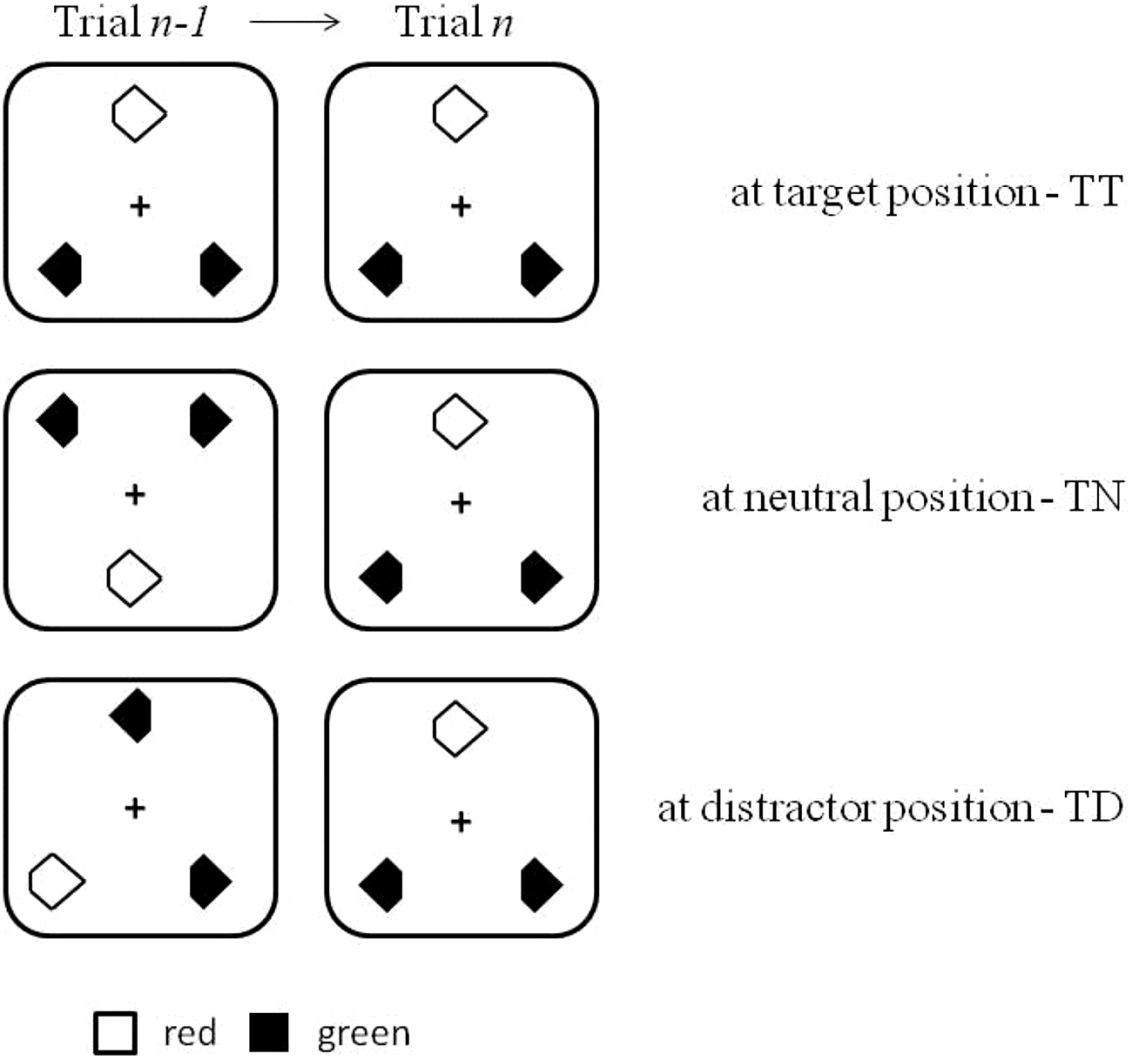
**Illustration of the search displays used in the current experiments.** Also shown is the location of the target across two successive trials: target at target location (TT), target at neutral, i.e., previously empty, location (TN), and target at distractor location (TD).

Mental reference frames can be conceptualized as (mnemonic) systems for encoding and maintaining item locations and/or the layout of objects in external space (Mou and McNamara, 2002). Reference systems may describe, or represent, object locations with regard to body coordinates (e.g., the coffee cup is located on my left-hand side – i.e., egocentric or *spatiotopic reference frame*); with regard to landmarks in the environment (e.g., the coffee cup is located in front of the monitor – i.e., allocentric or *object-centered reference frame*); or with regard to the eye coordinates (e.g., the coffee cup is located at that spot I look at – i.e., *retinotopic reference frame*). The present study focuses on the reference system(s) underlying the spatial representation of target and distractor items in short-term memory in ‘pop-out’ visual search (Maljkovic and Nakayama, 1996). In Maljkovic and Nakayama’s (1996) task, three search items (one target, two distractors) are presented in the form of a near-equilateral triangle (see **Figure 1**). The target is defined by a color difference relative to the distractors (e.g., a red target presented amongst green distractors, or vice versa, with randomized swapping of the target and distractor colors across trials). Observers’ task is to respond to the cut-off section of the color singleton target (left vs. right notch). The critical manipulation is the location of the target across trials. Observers respond faster when the target on a given trial (trial *n*) appears at a previous (trial *n-1*) target location relative to a neutral, that is, previously empty position, and they respond slower when the target appears at a previous distractor location. These ‘positional priming of pop-out’ effects are referred to as target location facilitation and distractor location inhibition, respectively, (Maljkovic and Nakayama, 1996). Subsequent work involving investigations of patients with left visual-field neglect (Finke et al., 2009) or imaging methods (i.e., event-related lateralized potentials; Gokce et al., 2014) showed that target location facilitation and distractor location inhibition are indeed independent effects (see also Gokce et al., 2013).

Based on these findings, the present study was designed to investigate two questions: (1) In what form are target and distractor locations in pop-out visual search represented in positional visual short-term memory (vSTM)? And (2) – assuming the existence of multiple reference frames – are there conditions that promote the use of one reference frame over the other in position priming? Several prior studies have already addressed the question of the reference frame(s) underlying positional vSTM (e.g., Maljkovic and Nakayama, 1996; Ball et al., 2009, 2010; Geyer et al., 2010; Tower-Richardi et al., 2012; Gokce, 2014). However, the results were mixed with regard to the exact reference frame(s) underlying performance. Arguably, therefore, in order to understand why the results and the conclusions drawn from these studies are divergent, it is important to look for any potentially crucial differences in the paradigms employed. This is the approach taken in the present study, namely, to re-investigate the reference frames underlying both target facilitation and distractor inhibition by systematically examining a set of different spatial manipulations in relation to positional priming effects.

### Multiple Reference Frames Support vSTM for Item Locations

In the recent years, a number of studies have elaborated on the beneficial role of visual memory (VM) for the guidance of visual search. Shore and Klein (2000) proposed to distinguish VM influences on visual search on various time scales, ranging from longer-lasting perceptual learning across blocks or sessions of trials (e.g., Chun and Jiang, 1998; Geyer et al., 2013) through VM influences across single experimental trials, that is, ‘cross-trial priming’ (e.g., Wang et al., 2005; Geyer et al., 2007), to VM influences within single trials, such as ‘inhibition of return’ (e.g., Müller and von Mühlenen, 2000; Klein et al., 2001) or ‘visual marking’ (e.g., Braithwaite et al., 2007; Müller et al., 2007).

Maljkovic and Nakayama (1996) were the first to show that positional cross-trial priming guides visual pop-out search. Using the three-item displays described above, they found that presentation of the trial *n* target at a trial *n-j* target location led to expedited reaction times (RTs), whereas presentation of the trial *n* target at a trial *n-j* distractor location led to slowed RTs (relative to presentation of the trial *n* target at a trial *n-j* empty, i.e., ‘neutral’ location). Maljkovic and Nakayama (1996) attributed these effects to an implicit memory (‘priming’) system whose function is to guide attention toward the location of the target and away from the stimuli recently avoided, that is, the distractors. These positional priming effects were evident across sequences of 5–8 preceding trials (Experiment 2) and cumulative, that is, the target location priming effect became larger as the number of repetitions increased (Experiment 3). Further, Maljkovic and Nakayama’s (1996) results are consistent with target location priming being supported by object-centered representations. In their Experiment 3, they presented the array of three search items in, across trials, systematically varying quadrants of the display screen. In this experiment, the three search items were arranged in a row rather than a triangle configuration, with the absolute position of the target (as well as of the distractors) being in one of the four display quadrants; the target’s relative position, by contrast, could be at the left, center, or right location within the item row. Across doublets of trials, both the target’s absolute and its relative position (both-same condition) or only its relative position in the row (relative-same condition) was repeated. For example, in the relative-same condition, the target appeared, say, at the center position of the row, but the absolute locations of the three search items changed across trials, for instance, from the top-left to the bottom-right display quadrant. Maljkovic and Nakayama (1996) found that target location priming was almost as large in the relative-same as in the both-same condition. They interpreted this finding as evidence for object-centered priming. One way in which object-centered priming may work is that observers use the overall ‘Gestalt’ of the three search items (the row or triangle configuration) for referencing target and distractor locations. Reference frames describing the positional relations of the search items may then be applied to the configural reference object (e.g., the triangle) and assist the search for the target and, importantly, the cross-trial tracking of search objects (an idea put forward by Geyer et al., 2010, in their relational-coding account). Another possibility, suggested by studies of multiple-object tracking, is that observers form combined representations centered on the mean location of the display items, rather than their individual locations (Alvarez and Oliva, 2008). The center-of-mass of the item locations may then serve as a referential anchor point.

However, other studies reported evidence that target locations are stored in positional priming memory in terms of a spatiotopic reference frame (Ball et al., 2009, 2010). Ball et al. (2009, 2010) used displays in which the search items were presented always in the center of the monitor. Observers had to discern the presence (vs. absence) of an orientation singleton: a left-tilted target line amongst right-tilted distractor lines (the display size was kept constant at 12 elements; a target was present in 80% of the trials). Target location priming was again assessed in both-same and relative-same conditions (in Ball et al.’s, 2009, 2010 terms: ‘allocentric’ and ‘retinotopic’ conditions). The main result was that target location priming was reliably stronger in the both-same than in the relative-same condition.

Taken together, the findings surveyed above suggest that multiple reference frames may be available to support location priming in visual search. This conclusion receives support also from other studies that used different paradigms. Examples include work on saccade planning (Pertzov et al., 2011), action selection (Keulen et al., 2002), relational (linguistic) descriptions (Carlson-Radvansky and Irwin, 1993), long-term spatial learning (Kelly and McNamara, 2010), and spatial perspective-taking (Mou and McNamara, 2002). However, the common question raised by all these studies, as well as those that used the positional priming paradigm, is what makes participants select one type of reference frame over another in a given experimental situation.

Arguably, one function of spatial reference frames is to aid visual search, including the cross-trial tracking of searched-for objects. In Maljkovic and Nakayama’s (1996) study, the search items were presented off-center and, across trials, in randomly varying screen quadrants. In contrast, Ball et al. (2009, 2010) presented the search items always in the center of the display (with the target’s absolute screen location varying, across trials, within the central array of search items). Accordingly, in the studies of Ball et al. (2009, 2010), it was certain for observers to expect, and subsequently search for, the target in the center of the display, whereas in Maljkovic and Nakayama’s (1996) study the items appeared in unpredictable quadrants across trials. This difference in the placement of the stimuli across trials may be the crucial factor responsible for the differential results, that is, the reference frame underlying positional priming may be influenced by certainty (or the lack thereof) with regard to the placement of the search items across trials (see also Kristjánsson et al., 2001). For example, when the relatively small, ‘local’ configuration of the three search items changes its overall, ‘global’ position randomly on the screen, while in a sense remaining the same configural (e.g., triangle) object, one may use an object-centered reference frame; in fact, under such conditions, a spatiotopic frame may not be available. In contrast, when the positioning of the local configuration is relatively invariant, then a spatiotopic frame can be used. Although an object-centered frame would also be available in this situation, a spatiotopic frame is selected primarily because it is possible to anticipate individual stimulus positions across trials and remap attention accordingly (this notion is developed further in the General Discussion).

On this background, the present study, consisting of two experiments, was designed to examine the effects of predictable versus unpredictable cross-trial item placements on the selection of reference frames in positional priming. Special emphasis was placed on the effects of distractor location inhibition, in addition to those of target location facilitation, as almost all prior studies had focused exclusively on the target location facilitation. For example, Maljkovic and Nakayama (1996) compared RT performance between targets presented at the position of a previous target versus a previous distractor item, thus being unable to separate the reference frames underlying target and distractor location priming. In other words, there was no ‘neutral’ baseline condition against which the effects of target presentation at previous target and, respectively, distractor locations could be compared. Similar arguments apply to recent fMRI investigations of positional priming (e.g., Geng et al., 2006; Kristjánsson et al., 2007; Rorden et al., 2011). Given this, the present experiments were devised to disentangle retinotopic, spatiotopic, and object-centered contributions to both target and distractor location priming.

**FIGURE 2:**
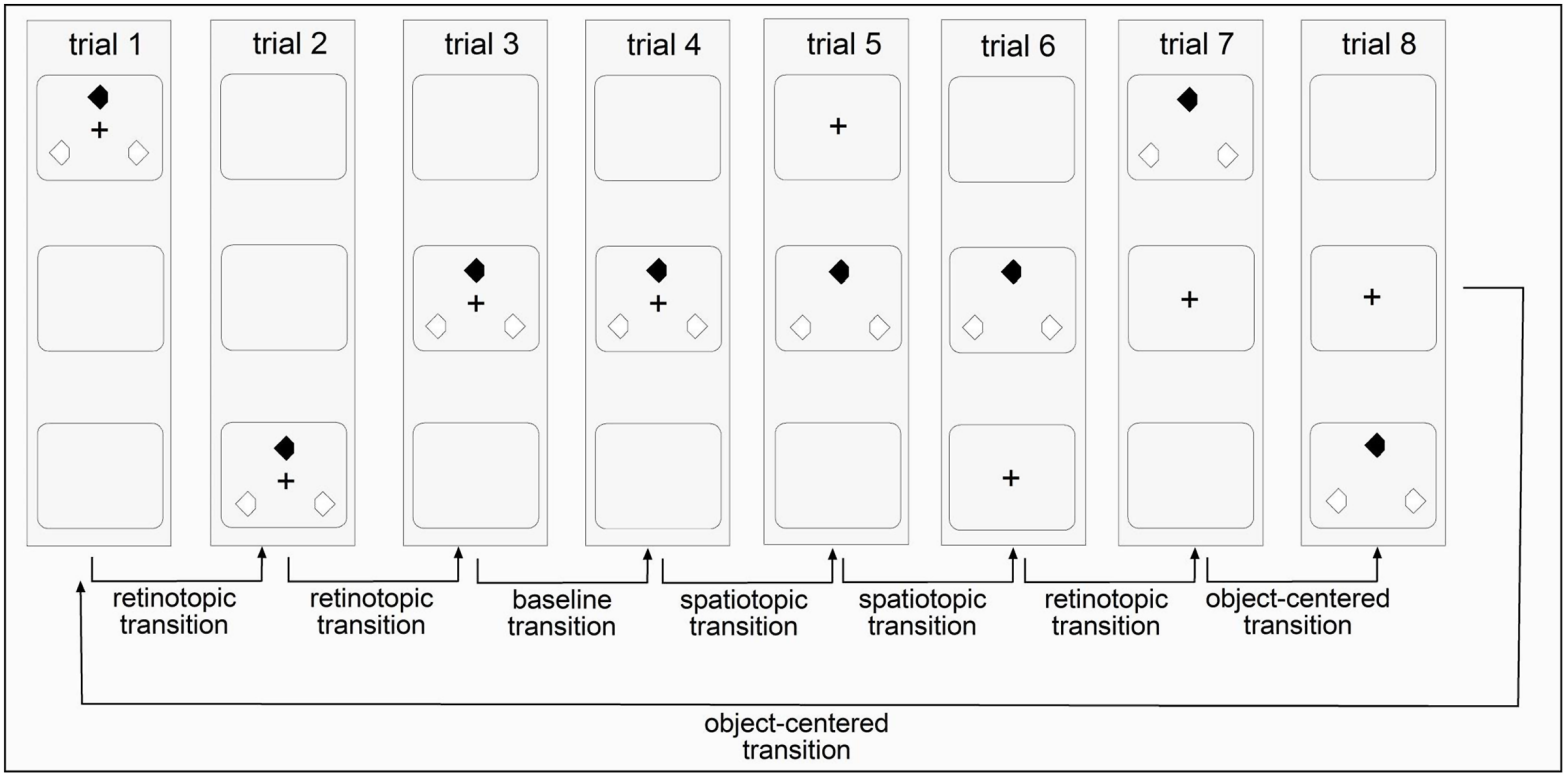
**Illustration of the four reference frame conditions in invariant sequences of Experiment 1 (vertical presentations).** Each trial is marked by an upright standing rectangle and the three squares inside the rectangle mark the three potential display regions for the fixation cross and the search items to appear. An invariant sequence contained eight trials and was repeatedly shown 126 times per session. In Experiment 2, the fixation cross and the search items were randomly displaced between the top, center, and bottom region across trials.

**FIGURE 3:**
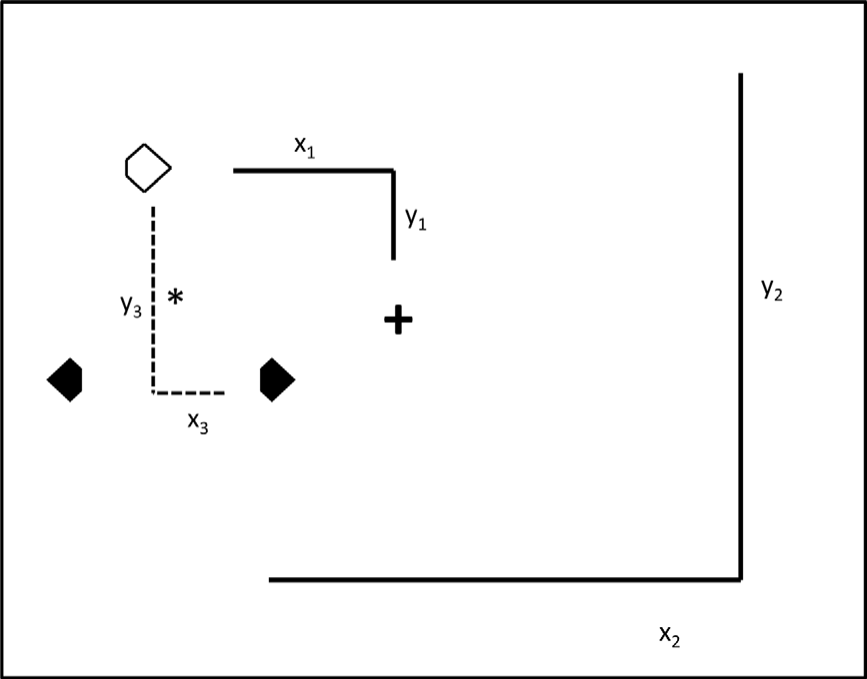
**Illustration of the three reference frames potentially supporting positional priming of pop-out search.** The large square represents the computer monitor. The spatial relationship between the search items in the ‘prime’ trial *n-1* and the ‘probe’ trial *n* were manipulated using variations of retinotopic, spatiotopic, and object-centered coordinates across pairs of trials. (1) Retinotopic reference frame: across doublets of trials, the search items remained at identical retinotopic positions, i.e., relative to the fixation cross (x_1_y_1_). (2) Spatiotopic reference frame: the three search items appeared at identical spatiotopic positions, i.e., relative to the computer monitor (x_2_y_2_), across the prime and probe trial. (3) Object-centered reference frame: here, the relations between the three search items remained constant in the display relative to the center-of-mass (*) (x_3_y_3_) across the two subsequent trials.

In addition to retinotopic, spatiotopic, and object-centered conditions, there was also an ‘all-repeat’ condition with a centrally presented search array which was meant to provide a ‘baseline’ measure for the maximum priming effects. In all other conditions, the search display ‘jumped’ between display regions arranged along the horizontal or vertical display axis. In the retinotopic condition, the fixation cross changed its position across trials, with the three search items changing their locations accordingly, ensuring that the retinotopic coordinates of the search items were kept constant across trials. In the spatiotopic condition, the fixation cross also changed its position across trials. However, the absolute positions of the search items and their spatiotopic coordinates were kept constant across trials (e.g., in the display center). In the object-centered condition, both the fixation cross and the search items changed their locations across trials in an independent fashion – in such a way that only object-centered information, that is, the triangular configuration of the search items, was kept constant across trials (see **Figures 2** and **3**).

The logic of these conditions was as follows: cross-trial priming effects should be largest in the baseline condition as all retinotopic, spatiotopic, and object-centered coordinates are repeated across trials (cf. Maljkovic and Nakayama, 1996; Ball et al., 2009, 2010; see also Barrett et al., 2003, for a similar rationale, albeit testing feature priming). By comparing cross-trial effects between the baseline and the other three conditions, one would be able to determine the reference frame(s) underlying target facilitation and distractor inhibition. For example, if priming effects are comparable between the baseline and the spatiotopic condition, but are reduced in the other conditions, then this would count as evidence for spatiotopic priming – because only in this and in the baseline condition would locational information, in terms of exact screen coordinates, be repeated across trials. In contrast, if positional priming is object-centered, then facilitation of target locations and/or inhibition of distractor locations should be equivalent between the baseline and all other conditions. This is because in all conditions, the three search items are ‘linked’ within the same triangular configuration (object) and this pattern would be repeated across trials.

## Materials and Methods

The general set-up was identical for Experiments 1 and 2. Participants performed a version of the priming of pop-out visual search task (Maljkovic and Nakayama, 1996). Across trials, the target-defining feature (color) and the response-defining feature (left/right orientation of the notch) were manipulated independently in addition to the location of the target (transition from preceding trial *n-1* to current trial *n*): target-at-target, target-at-distractor, or target-at-neutral location.

Each experiment consisted of two sessions in which the items were presented along the horizontal and vertical displays axes (order of conditions counter balanced across observers) across the consecutive trials. In Experiment 1, cross-trial item displacements followed an *invariant* (‘predictable’) sequence. In Experiment 2, in different sessions, search displays were presented in randomly chosen display regions across the horizontal or, respectively, vertical meridian (see **Figure 2**). Horizontal and vertical transitions were introduced to control for (minimize) the effects of visual factors on reference frame selection (Rubin et al., 1996; Laeng et al., 1998; Yeshurun and Carrasco, 1999; Carrasco et al., 2001; Corbett and Carrasco, 2011). For instance, Carrasco et al. (2001) found that detection performance across the visual field is inhomogeneous, with superior performance for targets positioned on the horizontal as compared to the vertical meridian. It is thus possible that positional priming differs across the horizontal versus vertical hemifields.

### Participants

Fourteen different observers participated in Experiments 1 and 2 (Experiment 1: female: 8; mean of age: 24 years, SD: 1 year; Experiment 2: female: 13; mean of age: 27 years, SD: 8 years). The observers were recruited from the subject panel of the Psychology Department (unit of General and Experimental Psychology /Neuro-Cognitive Psychology). All participants had normal or corrected-to-normal (color) vision and all but one were right-handed. They were naïve as to the purpose of the experiment, and informed afterwards about study aims. Participants gave written consent prior to their participation. Anonymity of their recorded and stored response data was guaranteed. Participants were paid at a rate of 8 Euro (10 USD) per hour or received course credits for their participation.

### Apparatus and Stimuli

Stimulus presentation and response recording were controlled by a standard Dell PC equipped with Microsoft Windows XP Prof operating system; the experimental control software was purpose-written in C++. An LG Plasma TV, with screen resolution set to 1440 × 900 pixels, was used for stimulus presentation. Participants responded via the computer keyboard placed in front of them. The distance between the participant and the screen was approximately 68 cm, with the viewing distance and head position maintained by the use of a chin rest. The experimental cabin was dimly lighted.

In both experiments, eye movements were monitored using an SR Research Eye Link II system (software version: 2.22), so that trials on which observers made saccades could be detected and excluded. **Table 1** presents the proportion of trials with accurate fixations separately for the two experiments. Eye movements were classified as saccades using Eye Link II’s standard settings (i.e., speed > 35/s; acceleration 9500/s^2^). A trial was considered as an eye movement trial if the eyes departed from the fixation cross and landed within an imaginary square frame, of side length 7.92°, centered on the search stimuli. A mixed-design ANOVA comparing the proportion of trials with eye movements across experiments (between-subject variable) and reference frame condition (within-subject variable) only revealed a main effect of the reference frame condition [*F*(3,78) = 14.50, *p* < 0.01, η^2^ = 0.22]: fixation accuracy was higher in the baseline (95%) than in the retinotopic, spatiotopic, and object-centered (93, 89, and 93%) conditions (all *p*’s < 0.01). Further, the stimuli were presented in the central region of a relatively large Plasma TV monitor (screen diagonal: 107 cm) to prevent edge effects on the determination of the reference frames used, such as cross-trial tracking of the search items relative to the monitor edges, which would be equivalent to operating in an object-centered frame.

**Table 1:** Fixation accuracies (%) in the four different spatial reference frame conditions of Experiments 1 and 2.

	Experiment 1 (%)	Experiment 2 (%)
Baseline	96	94
Retinotopic	94	93
Spatiotopic	89	88
Object-centered	94	92

The search display consisted of three diamond-shaped stimuli presented on a white background (30.0 cd/m^2^): one target and two distractors (size 2.21° × 1.88°). When the target was red, the distractors were green, and vice versa. The colors were chosen to be near-equiluminant: red, 7.7 cd/m^2^; green, 8.0 cd/m^2^. All stimuli had a cut-off section (size: 0.78° × 0.70°) either on the left or the right side, with side determined randomly (see **Figure 1**). The black fixation cross had a size of 1.49° × 1.34° and a luminance of 0.5 cd/m^2^. The fixation cross remained on the screen until the response was given, to make it easier for participants to suppress eye movements (i.e., they were instructed to maintain gaze at the fixation cross; see **Figure 2**). Pilot testing showed that with stimulus sizes of about 2.0° and cut-off section sizes of about 0.7° (the retinal eccentricity was approximately 10.0°), the target’s orientation could be discriminated without gaze shifts.

The search items (i.e., target and distractors) within the triangular configuration were arranged on an elliptical layout with horizontal and vertical axes of 7.14° and 6.77°, respectively. There were six possible target and distractor locations on the virtual ellipse. With respect to the previous trial *n-1*, targets on the current trial *n* appeared at one of three types of position: at the same position as the target on the previous trial (probability: 16%), at the position of a distractor on the previous trial (probability: 33%), or at a position where there had been no stimulus on the previous trial (i.e., neutral condition: probability: 50%). **Figure 1** illustrates the various cross-trial transition conditions.

In Experiments 1 and 2, the fixation cross and the search items were presented in three possible display regions. In different sessions, they were arranged either along the horizontal or the vertical display axis. In the horizontal sessions, the search items were presented in the left-center-right parts of the screen; and in the vertical session, they were presented in the top–center–bottom parts. The difference between the two experiments was in the regularity of the sequences for stimulus presentation. In Experiment 1, the appearance of the search items in a given display followed an invariant temporal structure: left → right → center → center → center → center → left → right (horizontal session) and top → bottom → center → center → center → center → top → bottom (vertical session). As can be seen, this structure consisted of eight ordered trials, which were repeated throughout the entire experiment (1.008/8 trials = 126 sequence repetitions in total). In a given sequence, both the positions of the search display (but not the target position) and of the fixation cross were predictable across trials. Note that although the placement of the search items followed a predictable sequence, observers could not predict the ‘local’ position of the target (within the triangular item configuration) on a given trial, and they were not expressly told about the sequence manipulation in Experiment 1. In Experiment 2, by contrast, the items appeared in a randomly chosen region: left → left → left → center → right →… (horizontal axis) and bottom → bottom → top → bottom → center →… (vertical axis). The particular sequence of search items was chosen such that (i) there was at least one transition in the baseline, spatiotopic, retinotopic, and object-centered conditions (see **Figure 2**) and (ii) that in these sequences, the number of trials in each reference frame condition was near-identical between the ‘predictable’ Experiment 1 and the ‘random’ Experiment 2 (18, 24, 18, 41% vs. 13, 25, 37, 25%, respectively; proportion of trials in the baseline, spatiotopic, retinotopic, and object-centered conditions). In both experiments, the display regions were separated by a center-to-center distance of 9.63°.

Observers’ task was to press the *Y*-key (on a German keyboard) when the target’s cut-off side was on the left and the *N*-key when the cut-off side was on the right, while responding as fast and accurately as possible. The dependent variables were: RTs, response errors, and oculomotor measures (i.e., saccade locations – used to detect and exclude critical eye movement trials).

### Procedure

Experiments 1 and 2 consisted of a practice session (three blocks of eight trials each; data not recorded) and two experimental sessions (horizontal and vertical presentations, 14 blocks × 72 trials each = 2.016 trials in total). In both experiments, the fixation cross was presented on a white background for 1000 ms and followed by the search items (one target, two distractors) which remained on the screen until the observers issued their response. The inter-trial interval was 1000 ms. No error feedback was given. Each experimental session lasted approximately 1.5 h.

### Design and Analyses

Across the two experiments, positional priming effects (target facilitation, distractor inhibition) were investigated in four different reference frame conditions: (1) baseline condition, (2) retinotopic condition, (3) spatiotopic condition, and (4) object-centered condition. In the *baseline* condition, the fixation cross and the three search stimuli were presented in identical regions (e.g., the center region) across two trials. This condition was intended to provide a full measure of positional priming, as the search stimuli appeared at the very same (retinotopic, spatiotopic, and object-centered) locations across trials. Note that the term ‘object’ here refers to the triangular configuration of the three search stimuli, which was identical across trials. In the *retinotopic* condition, the fixation cross and the search display would be located on, say, the left side of the screen on trial *n-1*, and on the right side on the subsequent trial (*n*). This would require participants to shift gaze from the left (i.e., the fixation cross on trial *n-1*) to the right (the fixation cross on trial *n*), and vice versa across other doublets of trials. Thus, in this condition only the retinotopic (and object-centered), but not the spatiotopic coordinates were identical across trials. In the *spatiotopic* condition, the fixation cross was located on, for example, the left side of the screen on trial *n-1* and the right side on trial *n*; but across the two trials, the search display was located in the center of the screen. Again, this condition would require participants to shift gaze from the left to the right (or vice versa), but the search items would appear at the very same screen coordinates across trials. Thus, in this condition only the spatiotopic (and object-centered), but not the retinotopic, information was repeated across trials. Finally, in the *object-centered* condition, the fixation cross was located, say, in the center of the screen across doublets of trials, but the search items changed absolute location from left to right and vice versa across trials. For example, when the items were located on the left side of the screen on trial *n-1*, then they were located on the right side on trial *n*. Importantly, in the object-centered condition, only information relating to the arrangement of the three search items (the triangle object), but neither the retinotopic nor the spatiotopic coordinates were repeated across the trials (see **Figure 3**).

## Results

Oculomotor data were pre-processed using SR Research’s ‘Data Viewer’ (version 1.8.221). Subsequent data analyses were performed using R (R Development Core Team, 2015) and SPSS (version 19). In all experiments, the first three (warm-up) trials in each block were excluded from the analysis. Further, the RT values outside the range ± 2.5 SD’s from the individual mean were discarded as outliers (Experiment 1: 2.7%; Experiment 2: 2.2%), in addition to eye movement (artifact) trials (see **Table 1**). For both experiments, data for each observer were collapsed across the horizontal and vertical sessions, as preliminary ANOVA’s had not revealed any effect involving the factor session^1^ (all *F*’s > 1). Moreover, error-response trials and trials following an error trial were also excluded from the analysis. Overall, search response accuracy was above 95% correct across all conditions (see **Table 2**). A mixed-design ANOVA of the error rates with the between-subject factor experiment (Experiments 1 and 2) and the within-subject factors target location (target at target, at neutral, at distractor location) and reference frame condition (baseline, retinotopic, spatiotopic, object-centered) revealed a significant effect of target position [*F*(2,52) = 5.20, MSE = 24.45, *p* < 0.01, η^2^ = 0.16]: fewer response errors occurred in the target-at-target and target-at-neutral relative to the target-at-distractor location condition (2.55, 2.83, 3.46%, respectively). As indicated by the significant three-way interaction [*F*(6,156) = 2.40, MSE = 6.62, *p* = 0.03, η^2^ = 0.08], this effect was most pronounced in the baseline condition of Experiment 2.

**Table 2:** Mean reaction times (RTs; in ms) and error rates (%) for trial *n* targets presented at trial *n-1* target (TT), neutral (TN), or distractor (TD) locations, separately for the baseline, retinotopic, spatiotopic, and object-centered conditions in Experiments 1 and 2.

	Experiment 1			Experiment 2		
	TT	TN	TD	TT	TN	TD
**Baseline**	578 (73); **3.5 (5.5)**	631 (89); **3.0 (2.8)**	659 (100); **3.9 (3.3)**	704 (74); **1.4 (1.9)**	758 (77); **3.3 (2.4)**	798 (72); **4.0 (2.8)**
**Retinotopic**	616 (88); **2.0 (2.3)**	647 (81); **3.0 (3.1)**	673 (86); **3.8 (3.5)**	690 (72); **2.6 (2.7)**	729 (69); **2.2 (1.5)**	753 (69); **3.2 (2.5)**
**Spatiotopic**	645 (76); **2.4 (2.5)**	686 (79); **2.7 (2.3)**	711 (92); **3.2 (2.8)**	721 (83); **2.4 (2.4)**	767 (70); **3.2 (2.5)**	803 (82); **3.0 (3.3)**
**Object-centered**	641 (74); **2.9 (3.5)**	660 (90); **2.8 (2.8)**	681 (93); **2.8 (2.4)**	736 (73); **3.1 (2.8)**	773 (72); **2.4 (1.5)**	792 (79); **3.8 (3.2)**

*SD for both mean RTs and error rates are reported in brackets.*

**FIGURE 4:**
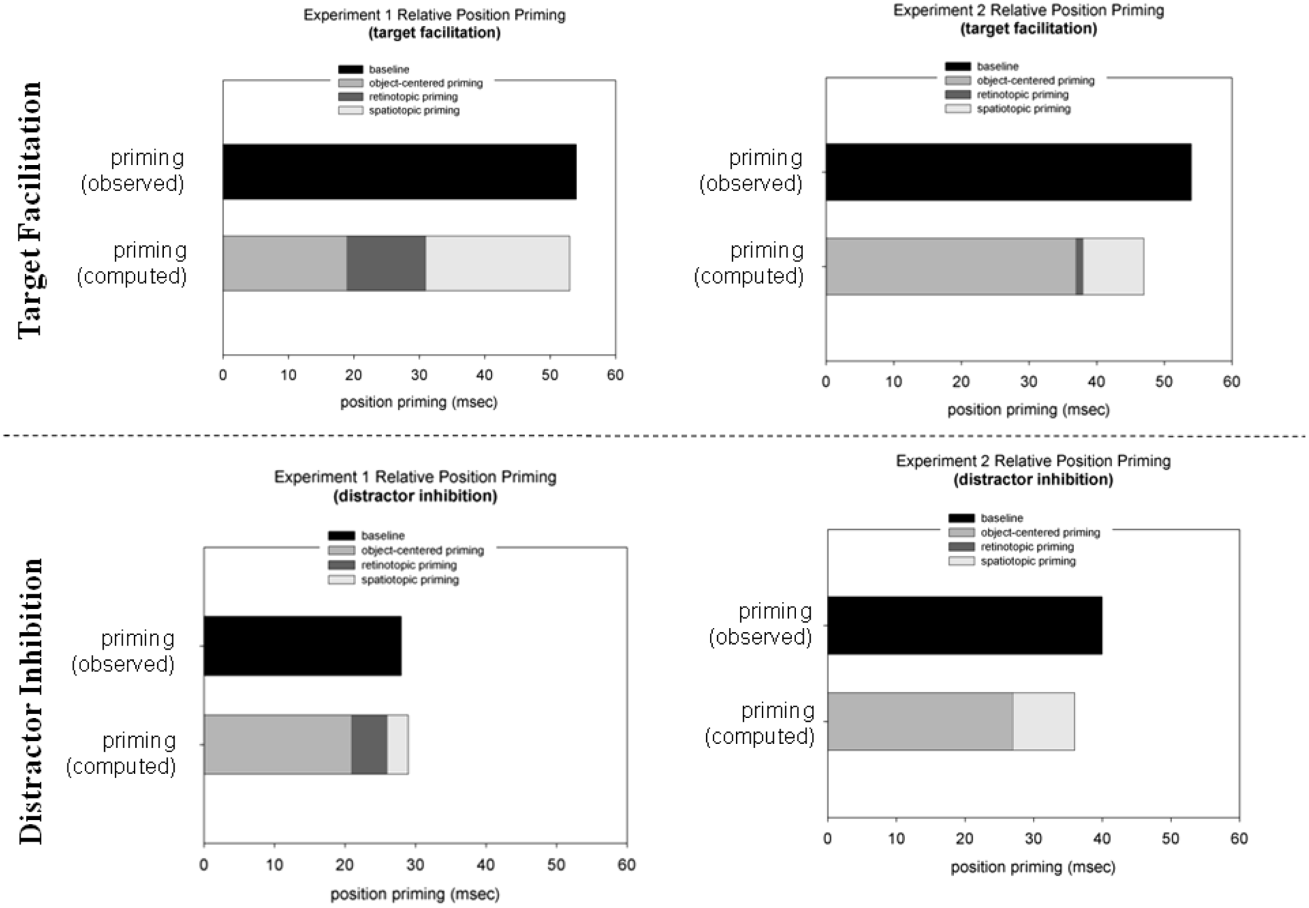
**Target location facilitation reaction time (RT target-at-target minus RT target-at-neutral location; upper panel; in ms) and distractor location inhibition (RT target-at-distractor minus RT target-at-neutral location; lower panel; in ms) in Experiments 1 (left panel) and 2 (right panel).** The black bars represent the obtained priming effects in the baseline condition. The gray bars represent (the sum of) the computed priming effects: spatiotopic priming –Δs (priming spatiotopic minus priming object-centered condition); retinotopic priming –Δr (priming retinotopic minus priming object-centered condition), and object-centered priming –Δo (priming in the object-centered condition).

Facilitatory and inhibitory position priming effects were examined by *post hoc* Tukey LSD tests^2^ (based on a separate, mixed-design ANOVA), comparing RTs to targets at target locations (facilitation) and, respectively, distractor locations (inhibition) relative to targets at neutral locations, for each experiment and reference frame condition. As shown in **Table 2** (see also **Figure 4**), the facilitatory effect was smaller in the object-centered and retinotopic conditions than in the baseline and spatiotopic conditions of Experiment 1 (19 and 31 ms vs. 54 and 41 ms; *p*’s < 0.05; the difference in priming effects between the retinotopic and spatiotopic condition was non-significant: 31 ms vs. 41 ms; *p* = 0.22) – whereas it was comparable across all reference frame conditions in Experiment 2 (38, 54, 38, and 47 ms; *p*’s > 0.10; data for the object-centered, baseline, retinotopic, and spatiotopic conditions, respectively). In contrast to facilitatory priming, inhibitory distractor location priming was comparable across the two experiments and reference frame conditions (Experiment 1: 21, 28, 26, 25 ms; Experiment 2: 27, 40, 25, 36 ms; *p*’s > 0.10; data for the object-centered, baseline, retinotopic, and spatiotopic conditions, respectively).

To recap our hypothesis, we expected to find differences in the representations of target and distractor locations between the ‘predictable’ Experiment 1 and ‘non-predictable’ Experiment 2. The results reported above show that facilitatory and inhibitory priming effects are indeed supported by multiple reference frames. Another way to look at the data is to compute the relative contributions of retinotopic, spatiotopic, and object-centered representations to priming in the baseline condition. According to the logic introduced above, the object-centered condition provides a pure measure of object-centered priming (Δo), because in this condition only information pertaining to the triangular configuration of the search items (the ‘object’) is repeated across trials. Spatiotopic (Δs) and retinotopic (Δr) priming can then be assessed by subtracting priming effects in the object-centered condition from priming effects in the spatiotopic and retinotopic conditions. Additivity of position priming would be reflected by the sum of the object-centered (Δo), spatiotopic (Δs), and retinotopic priming (Δr) being comparable to priming in the baseline condition^3^. The results of this analysis are presented in **Figure 4**. For Experiment 1, object-centered target location priming accounted for approximately 36% of priming in the baseline condition (retinotopic priming: ~23%; spatiotopic priming: ~41%). However, for Experiment 2, the contribution of object-centered target location priming increased to ~78% of total priming in the baseline condition (retinotopic priming: ~1%; spatiotopic priming: ~8%). A Pearson’s Chi-square test (with Yates continuity correction) comparing Δs and Δo between Experiments 1 and 2 revealed a significant result [χ^2^ = 20.06, *p* < 0.01]. By contrast, the object-centered component of inhibitory priming was relatively large overall, amounting to ~80% of priming in the baseline condition and uninfluenced by the predictable vs. unpredictable sequence manipulation (80 and 73% in Experiments 1 and 2, respectively; χ^2^ = 1.00, *p* = 0.32; see **Figure 4**).

One might argue that the contribution of an object-centered reference frame to positional priming was different across the two experiments simply due to Experiments 1 and 2 generating priming effects of different magnitude [with overall larger effects in Experiment 2; note that the experiment × target location interaction was significant (*F*(2,52) = 3.46, MSE = 1143.79, *p* = 0.03, η^2^ = 0.11)]. To test this, facilitatory and inhibitory priming were *z*-transformed (the means and SDs was taken from the baseline condition and the individual priming values from the other three conditions) and analyzed by means of a 2 (experiment) × 3 (zo, zr, zs) mixed-design ANOVA. This ANOVA revealed a significant interaction [*F*(2,52) = 3.74, *p* < 0.05, η^2^ = 0.19]. LSD *post hoc* tests confirmed that the amount of (normalized) object-centered target priming (zo) was larger in Experiment 2 than in Experiment 1 [-1.47 vs. -0.88; *p* = 0.04]. No differences were found for zr and zs between Experiments 1 and 2: zr: -1.00 and -0.91; zs: -0.83, and -0.92 (both *p*’s > 0.10). A similar analysis on normalized inhibitory distractor priming revealed only a main effect of the factor experiment [*F*(1,26) = 12,54, *p* < 0.01, η^2^ = 0.31], indicating that the inhibitory effect was overall larger in Experiment 2 than in Experiment 1 (-0,58 vs. -1,11; *p* < 0.01).

The above analysis suggests that object-centered contributions to target location priming are larger in Experiment 2. One idea of how object-centered priming may work is that observers encode the three search items as one ‘triangle’ ensemble and ‘pinpoint,’ that is, cross-trial track, individual locations with reference to the triangle object. If this is the case, then observers may eventually come to perceive the triangle object as rotating from trial to trial (see Geyer et al., 2007, whose observers had reported, in post-experimental debriefing, that they had indeed experienced the three search items as a triangle configuration rotating across trials). Applied to the present investigation, this could mean that even if the triangular configuration changed across trials (in the neutral condition), observers may have nevertheless been faster when the items’ relative positions were kept constant across trials. To examine this, we reanalysed RTs for targets at neutral positions (this was done across all reference frame conditions) according to whether two consecutively presented triangular configurations were rotationally (‘phenomenally’) the same or different across trials. In the ‘same’ condition, the two triangle configurations could be set into each other by a 180° rotation across trials. For example: on trial *n-1* the target was at the top-position of an upward-pointing triangle, while on trial *n* it was at the bottom-position of a downward-pointing triangle. In contrast, in the ‘different’ condition, two consecutive configurations were physically – and phenomenologically – different, for example: on trial *n-1* the target was at the top-position of an upward-pointing triangle, while on trial *n* it was at the top-left position of a downward-pointing triangle. If cross-trial tracking of individual locations is based on the items overall triangular configuration, and the rotation of the configuration across trials, RTs should be faster in the ‘same’ compared to the ‘different’ condition. Further, and given that Experiments 1 and 2 differed with regard to the contribution of object-centered representations (lower in Experiment 1 than 2), the difference between the ‘same’ and ‘different’ conditions should be more pronounced in the latter experiment. Statistically, we examined the two-way interaction of RTs in the neutral condition with the factors triangular configuration (phenomenally same, phenomenally different; within-subjects factor) and Experiments (1, 2; between-subjects factor). This analysis revealed a significant main effect of experiment [*F*(1,26) = 11.34, MSE = 416602.46, *p* < 0.05, η^2^ = 0.30], indicative of stronger cross-trial tracking of individual item positions with reference to the overall ‘Gestalt’ configuration in Experiment 2 (rotation-same vs. rotation-different conditions: 785 ms vs. 802 ms) than in Experiment 1 (673 ms vs. 686 ms).

Three additional analyses were conducted to examine the contribution of other (potentially ‘confounding’) factors in the determination of the pattern of position priming effects. First, in Experiment 1, the majority of trials (50%) were ‘center’ trials, making it possible that some types of priming were affected by this manipulation, in particular: retinotopic priming (recall that in Experiment 1, but not Experiment 2, priming was smaller in the retinotopic than in the spatiotopic/baseline conditions). Restated, differences in retinotopic priming may be owing to differences in the items’ retinal position (center vs. periphery) and these differences may come to the fore particularly under conditions of frequent center presentations. If so, then retinotopic priming should be smaller in transitions of central to peripheral (c → p) compared to peripheral to central (p → c) display presentations, particularly in the ‘center’ Experiment 1 (the critical transitions are given in **Figure 3**; the trial transitions concerned are trials 5 → 6 and trials 2 → 3, respectively). For target location facilitation, the Experiments (1, 2) × transition (c → p, p → c) mixed-design ANOVA revealed the main effects of transition [*F*(1,26) = 4.35, MSE = 18651.50, *p* = 0.04, η^2^ = 0.10] and experiment [*F*(1,26) = 5.32, MSE = 8850.28, *p* = 0.02, η^2^ = 0.05] to be significant. Interestingly, and contrary to the reasoning outlined above, target location facilitation was actually larger, and not smaller, for c → p relative to p → c transitions (Experiment 1: 41 ms vs. 30 ms; Experiment 2: 72 ms vs. 53 ms). No effect was found for distractor location inhibition (all *F*’s < 2). These results make it unlikely that differences in retinotopic priming are attributable to cross-experimental differences in the proportion of ‘center’ trials (50% vs. 33% in Experiments 1 and 2, respectively).

Second, we examined spatial compatibility effects relating to the display region of the stimuli and the response-critical target orientation (cut-off side) – an effect which may have exerted an influence particularly under conditions of horizontal display presentation^1^ (note, though, that no differences in the pattern of positional priming were found between the horizontal and vertical conditions). For example, spatial congruency of the (left/right) display region with the response-critical (left/right) cut-off side may facilitate the production of a left/right response, leading to overall faster RTs in the neutral condition and thus yielding diminished (enhanced) target (distractor) location priming. In an attempt to check this, we examined positional priming effects as a function of (in)compatibility between search display region (left, right) and the search task response (left button press, right button press) separately for target and distractor location priming. The results revealed no modulation of priming effects as a function of the congruency of the placement of the stimuli with the response-critical feature (target location priming, compatible trials: 32 and 36 ms; incompatible trials: 30 and 43 ms; distractor location priming, compatible trials: 26 and 36 ms; incompatible trials: 23 and 28 ms; all *p*’s > 0.05; data for Experiments 1 and 2, respectively).

Third, in addition to positional priming, we examined for potential effects of pop-out color priming (color repeated or swapped between target and distractors) and target orientation (repeated or changed), that is, response priming arising across two consecutive trials. Prior research had shown that position priming is largely independent of color priming (e.g., Maljkovic and Nakayama, 1996; Gokce et al., 2013), but may interact with response priming, particularly when response speed is low (e.g., Lamy et al., 2010). Given this, positional priming may be affected by repetitions versus changes of observers’ overt search task response. To examine this, RT performance was reanalyzed as a function of color and response repetition, in addition to the effects of target position, experiment, and reference frame. Color priming was calculated by subtracting RTs on same-color from RTs on different color-trials. Likewise, response priming was calculated by subtracting RTs on same-response from RTs on different-response trials. A mixed-design ANOVA with the factors target color (same, different), target response (same, different), target position (at target at neutral, at distractor location), Experiments (1, 2), and reference frame (baseline, retinotopic, spatiotopic, object-centered) revealed all main effects to be significant. Of most importance here are (i) the main effect of target color [*F*(1,26) = 1.61, MSE = 120058.16, *p* < 0.05, η^2^ = 0.02]: RTs were reliably faster for same- relative to different-colored targets (mean color priming in Experiments 1 and 2: 41 and 53 ms, respectively); (ii) the main effect of target (orientation) response [*F*(1,26) = 18.36, MSE = 8555.71, *p* < 0.05, η^2^ = 0.41]: RTs were faster for same- relative to different-response trials (mean response priming in Experiments 1 and 2: 5 and 17 ms, respectively); and (iii) a significant target position × target response interaction [*F*(2,52) = 2.66, MSE = 41794.80, *p* < 0.05, η^2^ = 0.66]. The interaction is due to the fact that the facilitatory priming effect was smaller for different-response compared to same-response trials in both experiments (Experiment 1: 32 ms vs. 76 ms; Experiment 2: 35 ms vs. 68 ms). Importantly, none of the ‘higher-order’ (4-way, 5-way) interactions involving the factors target color and/or target response were significant. These findings confirm that position and color priming are indeed independent phenomena, while also suggesting that position priming interacts with response priming (cf. Lamy et al., 2010). Importantly, though, repetitions versus changes of the target (orientation) response did not lead to qualitative changes in the pattern of positional priming effects in the four reference frame conditions of Experiments 1 and 2.

## Discussion

The current set of experiments investigated the spatial reference frames underlying positional priming of pop-out (Maljkovic and Nakayama, 1996). In two experiments, the three search items (one target, two distractors) appeared in various regions either along the horizontal or the vertical axis. In Experiment 1, the presentation of the search items followed an invariant temporal structure so that observers could predict the location of the upcoming search displays. In Experiment 2, by contrast, cross-trial item displacements were fully randomized so that observers could not predict the location of the upcoming search displays. It was found that target location priming was supported by both spatiotopic (Experiment 1) and object-centered representations (Experiment 2). Restated, the representation of target locations in cross-trial position memory varied as a function of the predictability of the sequences (see **Figure 2**). In contrast, distractor locations were encoded in position priming memory exclusively in an object-centered reference frame (Experiments 1 and 2). The multiplicity of the spatial reference frames underlying target location facilitation is in line with previous results (spatiotopic priming: Ball et al., 2009, 2010; object-centered priming: Maljkovic and Nakayama, 1996). However, it raises the question as to the cause of a transition from one reference frame to the other.

In the Introduction, we developed the hypothesis that the selection of a given spatial reference frame in position priming of pop-out search is contingent on the display factors, such as the predictability of the placement of the items across trials. A related idea is that positional priming is always supported by multiple – spatiotopic and object-centered – reference frames, but that their relative contributions to overall priming differ as a function of the predictability of the item placements. Based on an evaluation of previous investigations of the position priming task, the two experiments reported here tested the assumption that target location priming is more strongly supported by object-centered representations (Maljkovic and Nakayama, 1996) and that predictability can determine whether a spatiotopic reference frame can be used and, if so, to which extent it would contribute to positional priming (Ball et al., 2009, 2010). The results obtained were in line with these predictions.

To our knowledge, this is the first time that the effects of the predictability of item sequences have been shown to influence positional priming. Earlier studies have looked at the effects of predictability of repetitions on immediate position and feature priming effects (Maljkovic and Nakayama, 1994, 2000; Maljkovic and Martini, 2005; Geyer and Müller, 2009). These studies typically show that re-presentation of the target at a previous location (or, respectively, re-presentation of the target color) across longer sequences of trials leads to larger, that is, cumulative, priming effects. Geyer and Müller (2009) found that priming increased with each stimulus repetition and that this increase was larger when position/color repetitions occurred on the majority of trials and, thus, were expected (they compared priming effects in this high-repeat condition with priming effects in a baseline condition in which position/color repetitions vs. changes were equally likely – importantly, priming effects in the two conditions were compared between identical sequences of repeat trials). They interpreted this result as evidence for an effect of top–down expectancy on color and position priming. However, the transition of target location priming from an object-centered to a spatiotopic reference frame as a function of expectation is a novel finding and adds to the existing evidence on top–down controlled priming.

For example, Summerfield et al. (2008) compared responses to repeated and non-repeated face stimuli measured by functional magnetic resonance imaging (fMRI). The important finding was that of reductions of blood oxygen level-dependent (BOLD) responses on repeated compared to non-repeated trials (i.e., repetition suppression, Grill-Spector et al., 2006) being larger when repetitions were frequent and thus expected. In a follow-up study, Larsson and Smith (2012) also found reductions in the BOLD responses for expected stimuli, provided that the observers attended to the face stimuli (but see Stefanics et al., 2011, for evidence of top–down modulated priming in the absence of covert attention). More recently, Summerfield et al. (2011) replicated their original finding using more direct, that is, temporally precise, electroencephalographic (EEG) measures (the result was that of enhanced ERP amplitudes, approximately 300 ms after stimulus onset at central electrodes, for repeated relative to non-repeated trials when repetitions occurred in the majority of trials). Summerfield et al. (2011) took their results to mean that predictive-coding models (Friston, 2010) may provide an appropriate account for repetition priming. However, it is worth noting that the evidence for a top–down view of priming was limited to neural (fMRI, EEG) measures and the processing of highly trained stimuli (faces). On this background, the present finding of top–down controlled priming in visual search for artificial, ‘laboratory’ stimuli would further strengthen the idea that priming, including behavioral priming, is at least in part a top–down effect.

In the present investigation, expectation effects were reflected in differences in the reference frames underlying target location priming in invariant versus random sequences. Interestingly, informal questioning of observers after the ‘predictive’ Experiment 1 revealed that only 6 out of the 14 were aware of the sequential manipulation. Further, target location priming in the spatiotopic condition of Experiment 1 did not differ between ‘aware’ and ‘unaware’ participants [33 ms vs. 31 ms; *t*(12) = 0.18, *p* = 0.42]. Therefore, the selection of a certain reference frame seems to occur automatically (see, e.g., Carlson-Radvansky and Irwin, 1993, for a similar argument albeit looking at reference frame selection in linguistic descriptions of scenes). With respect to the top–down view of priming, this would mean that although invariant sequences are learned and subsequently guide the selection of single reference frame, observers are nevertheless not aware of their learning. Given this, the present results may be considered as evidence for *implicit top–down priming* (cf. Wolfe et al., 2003).

### Configural Processing in Positional Priming and Spatial Working Memory

In the current study, we put forward the thesis that predictability – or the lack thereof – of item sequences is an important factor in the determination of target location reference frames. Support for this claim comes also from other studies that have investigated the representation(s) underlying the maintenance of multiple item locations in visual working memory across shorter and longer time spans (e.g., Chun and Jiang, 1998; Jiang et al., 2000; Boduroglu and Shah, 2009; Golomb and Kanwisher, 2012; Gokce, 2014). As the present study is similar to some of these, it is worth to compare the current repetition effects to recent work, in particular, on the organization of spatial working memory (Jiang et al., 2000; Gmeindl et al., 2011). A review of these studies suggests that spatial working memory and positional priming share common mechanisms (considered further below). For example, Gmeindl et al. (2011) showed that the representation of a single stimulus location in working memory is influenced by the locations of the surrounding items. In their Experiment 2, observers performed a location change detection task. The memory and probe displays contained three items, arranged in a virtual triangle. This configuration could be either repeated or changed: in the latter condition, two of the three items were displaced in random directions in the test display (the memory and test displays were separated by a gap of 3000 ms). Observers’ task was to indicate whether a probe item appeared at the location of a target item (the critical, to-be-judged stimulus was presented in red color; the other items were black). It was found that observers exhibited higher accuracy on location match, compared to non-match, trials. Furthermore, for non-match trials, observers’ performance was higher for trials with changed relative to repeated configurations. Gmeindl et al. (2011) took this result to mean that target location detection is influenced by configural information, specifically, that configural attributes aid information processing at a decision stage where evidence for a mismatch between item locations in the memory and test displays is evaluated – the idea being that detection of a change in stimulus locations is enhanced when the configuration changes, too. This builds upon evidence, reported by Boduroglu and Shah (2009; see also Jiang et al., 2000 or Hyun et al., 2009), that observers, in WM tasks, encode both task-relevant (here: item locations) and task-irrelevant (here: item configuration) information and that the latter can bias observers’ (location) change detection performance. Applied to the present visual pop-out search task, this could mean that in default mode, observers maintain item locations by means of a configuration-dependent (object-centered) code in positional short-term memory (Experiment 2). The reason for this might be that configuration-based coding, or ‘Gestalt’ grouping, can reduce memory load, particularly in the cross-trial tracking of distractor positions. This idea builds on the assumption that observers intend to reduce memory load in positional priming, as they do in WM tasks.

Additionally, saliency of target features can also reduce VM load. For example, using a condition in which both the target and probe item were red color singletons, Gmeindl et al. (2011) found that target location detection performance was uninfluenced by changes of the item configuration. In this case, WM might be supported by location-specific representations. As regards the present pop-out task, with one salient target and two distractors, it is possible that observers are able to segregate relevant from non-relevant information particularly under conditions of predictable item displacements and maintain target locations by means of a configuration-independent (spatiotopic) code in positional vSTM.

An alternative, though not mutually exclusive, view is to consider performance with predictable cross-trial item sequences as being supported by predictive attention mechanisms, aiding selective processing to keep track of the ‘relevant’ items across shifts on the retina (e.g., Rolfs et al., 2011; Jonikaitis et al., 2013). In Jonikaitis et al. (2013), observers had to make a saccade to a certain location, while discriminating the orientation of a briefly presented Gabor patch at another, spatially cued location, including but not limited to the saccade location. Interestingly, Jonikaitis et al. (2013) found that some 100 ms before the onset of the saccade, probe discrimination performance was best at the spatial location of the Gabor patch after the saccade, suggesting anticipatory remapping of spatiotopic attention to relevant (i.e., cued) items. In the present investigation of memory-based guidance of attention, observers had to shift gaze to predictable locations (regions) across trials, rather than within trials (as in Jonikaitis et al., 2013), so that anticipatory remapping could have aided pop-out search only across trials. (If this was indeed the case, it would mean that cross-trial effects complement the beneficial effects of remapping within trials!) Conceivably, remapping is influenced by the statistical structure of the search environment. Recall that in Experiment 1, the placement of the search arrays and the fixation crosses were entirely predictable across trials. Observers may have implicitly learned these regularities, forming associations between the global position of the fixation cross and the search items across trials. These contingencies may then have served as cues for cross-trial remapping.

In sum, target position priming may, in default mode, be supported by object-centered representations and predictability of stimulus placements may engage a transition to a spatiotopic reference frame – or up-modulate the relative contribution of spatiotopic to object-centered representations – in target position priming. One conceivable mechanism of how spatiotopic priming could work is through anticipatory remapping of attention towards memorized target locations.

### Working Memory = Positional Priming?

Visual priming may be considered as reflecting a form of implicit sensory memory that automatically buffers information for the task at hand. Working memory, by contrast, is a system that actively maintains information for a given task. Although there is good evidence that the two forms of memory reflect qualitatively different phenomena, a number of recent studies suggest that priming and working memory nevertheless share functions and neural resources. This idea is consistent with investigations of the brain structures underlying priming and working memory, showing that biasing signals from both types of memory modulate activity in the same brain areas (i.e., visuo-cortical areas V1 and V2; see Soto et al., 2007, 2012). Other studies using behavioral measures have demonstrated that priming effects are modulated by the addition of a secondary working memory task. For example, Geyer et al. (2011) showed that the maintenance of a triangular shape in working memory enhanced positional priming in three-item ‘triangle’ displays. Similarly, Kristjánsson et al. (2013) found that featural priming was attenuated when observers had to maintain featural (i.e., color) information in working memory. Kristjánsson et al. (2013) took the latter to mean that working memory and priming, in addition to selective attention, are supported by a common resource pool (see also Kristjánsson et al., 2007; Anderson et al., 2013). Given these demonstrations, it is well possible that similar principles apply to the storage of configural information in positional priming and spatial working memory.

## Conclusion

In summary, the current study supports the view that multiple reference frames are simultaneously available to positional priming of pop-out search. Target location priming is supported by both spatiotopic and object-centered reference frames, whereas distractor location priming is supported only by object-centered representations. We suggest that predictability of the item sequences modulate the transition – or relative weight – of one reference frame over the other for target location priming.

## Conflict of Interest Statement

The authors declare that the research was conducted in the absence of any commercial or financial relationships that could be construed as a potential conflict of interest.

## Footnotes

1 Separate mixed-design ANOVAs for horizontal and vertical sequences showed that for horizontal sequences, there were main effects of target position [*F*(2,52) = 403.87, MSE = 121857.23 *p* < 0.05, η2 = 0.94] and reference frame condition [*F*(3,78) = 37.71, MSE = 22455.94, *p* < 0.05, η2 = 0.59], in addition to a significant experiment × reference frame interaction [*F*(3,78) = 13.44, MSE = 8004.11, *p* < 0.05, η2 = 0.34]. For vertical sequences, the main effects of target position [*F*(2,52) = 198.71, MSE = 136984.42, *p* < 0.05, η2 = 0.88] and reference frame condition [*F*(3,78) = 47.36, MSE = 36731.25, *p* < 0.05, η2 = 0.64] were significant. The experiment × reference frame interaction also reached significance [*F*(3,78) = 22.05, MSE = 17101.38, *p* < 0.05, η2 = 0.45].

2 The mixed-design ANOVA with the between-subject factor Experiments (1, 2), the within-subject factors target position (at neutral, at target, at distractor position), and the within-subject factor reference frame (baseline, retinotopic, spatiotopic, object-centered) revealed significant main effects of target location [*F*(2,52) = 390.46, MSE = 129088.36, *p* < 0.001, η2 = 0.93] and reference frame condition [*F*(3,78) = 69.85, MSE = 28975.81, *p* < 0.001, η2 = 0.72]. Further, the experiment × target position interaction [*F*(2,52) = 3.46, MSE = 1143.79, *p* = 0.03, η2 = 0.11] and the reference frame × target position interaction [*F*(6,156) = 10.21, MSE = 2106.35, *p* < 0.001, η2 = 0.28] were significant. The experiment × reference frame × target position interaction approached significance [*F*(6,156) = 1.73, *p* = 0.11, η2 = 0.09].

3 Note that additional correlation analysis revealed evidence for the independence of position priming effects (see also Maljkovic and Nakayama, 1996; Tower-Richardi et al., 2012). Mean correlations for target and, respectively, distractor, location priming in the retinotopic, spatiotopic, and object-centered reference frame conditions ranged between −0.18 and 0.18 (Spearman’s R), with none of the correlations reaching significance (all *p*’s > 0.49). Note, though, that this finding is to be taken with caution, as sample sizes were small and the associated 95% confidence interval broad (with R ranging from –0.64 to +0.64).

## References

[B1] Alvarez G. A. Oliva A. (2008). The representation of simple ensemble visual features outside the focus of attention. *Psychol. Sci.* 19 392–398. 18399893 10.1111/j.1467-9280.2008.02098.x PMC2587223

[B2] Anderson D. E. Vogel E. K. Awh E. (2013). A common discrete resource for visual working memory and visual search. *Psychol. Sci.* 24 929–938. 23572280 10.1177/0956797612464380 PMC4450974

[B3] Ball K. Smith D. Ellison A. Schenk T. (2009). Both egocentric and allocentric cues support spatial priming in visual search. *Neuropsychologia* 47 1585–1591. 19084545 10.1016/j.neuropsychologia.2008.11.017

[B4] Ball K. Smith D. Ellison A. Schenk T. (2010). A body-centered frame of reference drives spatial priming in visual search. *Exp. Brain Res.* 204 585–594. 20574687 10.1007/s00221-010-2327-y

[B5] Barrett D. J. K. Bradshaw M. F. Rose D. (2003). Endogenous shifts of covert attention operate within multiple coordinate frames: evidence from a feature-priming task. *Perception* 32 41–52. 12613785 10.1068/p3298

[B6] Boduroglu A. Shah P. (2009). Effects of spatial configurations on visual change detection: an account of bias changes. *Mem. Cognit.* 37 1120–1131. 10.3758/MC.37.8.1120 19933456

[B7] Braithwaite J. J. Humphreys G. W. Hulleman J. Watson D. G. (2007). Fast color grouping and slow color inhibition: evidence for distinct temporal windows for separate processes in preview search. *J. Exp. Psychol. Hum. Percept. Perform.* 33 503–517. 17563218 10.1037/0096-1523.33.3.503

[B8] Carlson-Radvansky L. A. Irwin D. E. (1993). Frames of reference in vision and language: where is above? *Cognition* 46 223–244. 8462273 10.1016/0010-0277(93)90011-j

[B9] Carrasco M. Talgar C. P. Cameron E. L. (2001). Characterizing visual performance fields: effects of transient covert attention, spatial frequency, eccentricity, task and set size. *Spat. Vis.* 15 61–75. 11893125 10.1163/15685680152692015 PMC4332623

[B10] Chun M. M. Jiang Y. (1998). Contextual cueing: implicit learning and memory of visual context guides spatial attention. *Cogn. Psychol.* 36 28–71. 9679076 10.1006/cogp.1998.0681

[B11] Corbett J. E. Carrasco M. (2011). Visual performance fields: frames of reference. *PLoS ONE* 6:e24470 10.1371/journal.pone.0024470 PMC3169603 21931727

[B12] Finke K. Bucher L. Kerkhoff G. Keller I. von Rosen F. Geyer T. (2009). Inhibitory and facilitatory location priming with left-sided visual hemi-neglect. *Psychol. Res.* 73 177–185. 19066947 10.1007/s00426-008-0209-8

[B13] Friston K. (2010). The free-energy principle: a unified brain theory? *Nat. Rev. Neurosci.* 11 127–138. 20068583 10.1038/nrn2787

[B14] Geng J. J. Eger E. Ruff C. C. Kristjánsson A. Rotshtein P. Driver J. (2006). On-line attentional selection from competing stimuli in opposite visual fields: effects on human visual cortex and control processes. *J. Neurophysiol.* 96 2601–2612. 16855105 10.1152/jn.01245.2005

[B15] Geyer T. Gokce A. Müller H. J. (2011). Reinforcement of inhibitory positional priming by spatial working memory contents. *Acta Psychol.* 137 235–242. 10.1016/j.actpsy.2010.06.009 20624618

[B16] Geyer T. Müller H. J. (2009). Distinct, but top-down modulable color and positional priming mechanisms in visual pop-out search. *Psychol. Res.* 73 167–176. 19082623 10.1007/s00426-008-0207-x

[B17] Geyer T. Müller H. J. Assumpcao L. Gais S. (2013). Sleep-effects on implicit and explicit memory in repeated visual search. *PLoS ONE* 8:e69953 10.1371/journal.pone.0069953 PMC3732254 23936363

[B18] Geyer T. Müller H. J. Krummenacher J. (2007). Cross-trial priming of element positions in pop-out visual search is dependent on stimulus arrangement. *J. Exp. Psychol. Hum. Percept. Perform.* 33 788–797. 17683228 10.1037/0096-1523.33.4.788

[B19] Geyer T. Zehetleitner M. Müller H. J. (2010). Positional priming of pop-out-out: a relational-encoding account. *J. Vis.* 10 1–17. 20462304 10.1167/10.2.3

[B20] Gmeindl L. Nelson J. K. Wiggin T. Reuter-Lorenz P. A. (2011). Configural representations in spatial working memory: modulation by perceptual segregation and voluntary attention. *Atten. Percept. Psychophys.* 73 2130–2142. 10.3758/s13414-011-0180-0 PMC3205194 21761373

[B21] Gokce A. (2014). *Configuration**-Centered Positional Priming of Visual Pop-Out Search*. Ph.D. thesis, Ludwig-Maximilians-Universität München, Munich.

[B22] Gokce A. Geyer T. Finke K. Müller H. J. Töllner T. (2014). What pops out in positional priming of pop-out: insights from event-related EEG lateralizations. *Front. Psychol.* 5:688 10.3389/fpsyg.2014.00688 PMC4078256 25071658

[B23] Gokce A. Müller H. J. Geyer T. (2013). Positional priming of pop-out is nested in visuospatial context. *J. Vis.* 13:32. 10.1167/13.3.32 24281321

[B24] Golomb J. D. Kanwisher N. (2012). Retinotopic memory is more precise than spatiotopic memory. *Proc. Natl. Acad. Sci.* 109 1796–1801. 22307648 10.1073/pnas.1113168109 PMC3277191

[B25] Grill-Spector K. Henson R. Martin A. (2006). Repetition and the brain: neural models of stimulus-specific effects. *Trends Cogn. Sci.* 10 14–23. 16321563 10.1016/j.tics.2005.11.006

[B26] Hyun J. S. Woodman G. F. Vogel E. K. Hollingworth A. Luck S. J. (2009). The comparison of visual working memory representations with perceptual inputs. *J. Exp. Psychol. Hum. Percept. Perform.* 35 1140–1160. 19653755 10.1037/a0015019 PMC2726625

[B27] Jiang Y. Olson I. R. Chun M. M. (2000). Organization of visual short-term memory. *J. Exp. Psychol. Learn. Mem. Cogn.* 26 683–702. 10855426 10.1037//0278-7393.26.3.683

[B28] Jonikaitis D. Szinte M. Rolfs M. Cavanagh P. (2013). Allocation of attention across saccades. *J. Neurophysiol.* 109 1425–1434. 23221410 10.1152/jn.00656.2012

[B29] Kelly J. W. McNamara T. P. (2010). Reference frames during the acquisition and development of spatial memories. *Cognition* 116 409–420. 20591422 10.1016/j.cognition.2010.06.002 PMC2914168

[B30] Keulen R. F. Adam J. J. Fischer M. H. Kuipers H. Jolles J. (2002). Selective reaching: evidence for multiple frames of reference. *J. Exp. Psychol. Hum. Percept. Perform.* 28 515–526. 12075885

[B31] Klein R. M. Munoz D. P. Dorris C. Taylor T. L. (2001). Inhibition of return in monkey and man. *Adv. Psychol.* 133 27–47.

[B32] Kristjánsson Á. Mackeben M. Nakayama K. (2001). Rapid, object-based learning in the deployment of transient attention. *Perception* 30 1375–1387. 11768490 10.1068/p3251

[B33] Kristjánsson Á. Saevarsson S. Driver J. (2013). The boundary conditions of priming of visual search: from passive viewing through task-relevant working memory load. *Psychon. Bull. Rev.* 20 514–521. 10.3758/s13423-013-0375-6 23325704

[B34] Kristjánsson A. Vuilleumier P. Schwartz S. Macaluso E. Driver J. (2007). Neural basis for priming of pop-out during visual search revealed with fMRI. *Cereb. Cortex* 17 1612–1624. 16959868 10.1093/cercor/bhl072 PMC2600429

[B35] Laeng B. Peters M. McCabe B. (1998). Memory for locations within regions: spatial biases, and visual hemifield differences. *Mem. Cogn.* 26 97–107. 10.3758/bf03211373 9519700

[B36] Lamy D. Yashar A. Ruderman L. (2010). A dual-stage account of inter-trial priming effects. *Vis. Res.* 50 1396–1401. 20079758 10.1016/j.visres.2010.01.008

[B37] Larsson J. Smith A. T. (2012). fMRI repetition suppression: neuronal adaptation or stimulus expectation? *Cereb. Cortex* 22 567–576. 21690262 10.1093/cercor/bhr119 PMC3278317

[B38] Maljkovic V. Martini P. (2005). Implicit short-term memory and event frequency effects in visual search. *Vision Res.* 45 2831–3846. 16023172 10.1016/j.visres.2005.05.019

[B39] Maljkovic V. Nakayama K. (1994). Priming of pop-out I: role of features. *Mem. Cognit.* 22 657–672. 10.3758/bf03209251 7808275

[B40] Maljkovic V. Nakayama K. (1996). Priming of pop-out-out II. The role of position. *Percept. Psychophys.* 58 977–991. 8920835 10.3758/bf03206826

[B41] Maljkovic V. Nakayama K. (2000). Priming of pop-out III. A short term implicit memory system beneficial for rapid target selection. *Vis. Cogn.* 7 571–595.

[B42] Mou W. McNamara T. P. (2002). Intrinsic frames of reference in spatial memory. *J. Exp. Psychol. Learn. Mem. Cogn.* 28 162–170. 11827078 10.1037/0278-7393.28.1.162

[B43] Müller H. J. von Mühlenen A. (2000). Probing distractor inhibition in visual search: inhibiton of return. *J. Exp. Psychol. Hum. Percept. Perform.* 26 1591–1605. 11039487 10.1037//0096-1523.26.5.1591

[B44] Müller H. J. von Mühlenen A. Geyer T. (2007). Top-down inhibition of search distractors in parallel visual search. *Percept. Psychophys.* 69 1373–1388. 18078228 10.3758/bf03192953

[B45] Pertzov Y. Avidan G. Zohary E. (2011). Multiple reference frames for saccadic planning in the human parietal cortex. *J. Neurosci.* 31 1059–1068. 21248131 10.1523/JNEUROSCI.3721-10.2011 PMC6632924

[B46] R Development Core Team. (2015). *R: A Language and Environment for Statistical Computing.* Vienna: R Foundation for Statistical Computing.

[B47] Rolfs M. Jonikaitis D. Deubel H. Cavanagh P. (2011). Predictive remapping of attention across eye movements. *Nat. Neurosci.* 14 252–256. 21186360 10.1038/nn.2711

[B48] Rorden C. Kristjansson A. Revill K. P. Saevarsson S. (2011). Neural correlates of inter-trial priming and role-reversal in visual search. *Front. Hum. Neurosci.* 5:151 10.3389/fnhum.2011.00151 PMC3228257 22144956

[B49] Rubin N. Nakayama K. Shapley R. (1996). Enhanced perception of illusory contours in the lower versus upper visual hemifields. *Science* 271 651–653. 8571128 10.1126/science.271.5249.651

[B50] Shore D. I. Klein R. M. (2000). On the manifestations of memory in visual search. *Spat. Vis.* 14 59–75. 11334182 10.1163/156856801741369

[B51] Soto D. Humphreys G. W. Rotshtein P. (2007). Dissociating the neural mechanisms of memory-based guidance of visual selection. *Proc. Natl. Acad. Sci.* 104 17186–17191. 17940037 10.1073/pnas.0703706104 PMC2040391

[B52] Soto D. Llewelyn D. Silvanto J. (2012). Distinct causal mechanisms of attentional guidance by working memory and repetition priming in early visual cortex. *J. Neurosci.* 32 3447–3452. 22399767 10.1523/JNEUROSCI.6243-11.2012 PMC6621055

[B53] Stefanics G. Kimura M. Czigler I. (2011). Visual mismatch negativity reveals automatic detection of sequential regularity violation. *Front. Hum. Neurosci.* 5:46 10.3389/fnhum.2011.00046 PMC3099311 21629766

[B54] Summerfield C. Trittschuh E. H. Monti J. M. Mesulam M. Egner T. (2008). Neural repetition suppression reflects fulfilled perceptual expectations. *Nat. Neurosci.* 11 1004–1006. 19160497 10.1038/nn.2163 PMC2747248

[B55] Summerfield C. Wyart V. Johnen V. M. de Gardelle V. (2011). Human scalp electroencephalography reveals that repetition suppression varies with expectation. *Front. Hum. Neurosci.* 5:67 10.3389/fnhum.2011.00067 PMC3147224 21847378

[B56] Tower-Richardi S. Leber A. Golomb J. (2012). Priming of pop-out is preserved across eye movements. *J. Vis.* 12:1250.

[B57] Wang D. Kristjánsson Á. Nakayama K. (2005). Efficient visual search without top-down or bottom-up guidance. *Percept. Psychophys.* 67 239–253. 15971688 10.3758/bf03206488

[B58] Wolfe J. M. Butcher S. J. Lee C. Hyle M. (2003). Changing your mind: on the contributions of top-down and bottom-up guidance in visual search for feature singletons. *J. Exp. Psychol. Hum. Percept. Perform.* 29 483–502. 12760630 10.1037/0096-1523.29.2.483

[B59] Yeshurun Y. Carrasco M. (1999). Spatial attention improves performance in spatial resolution tasks. *Vision Res.* 39 293–306. 10326137 10.1016/s0042-6989(98)00114-x

